# Artificial intelligence in cancer risk assessment of oral potentially malignant disorders: applications and challenges

**DOI:** 10.1097/JS9.0000000000003363

**Published:** 2025-09-08

**Authors:** Yan Jiao, Yu-Jiao Lin, Tong Liu

**Affiliations:** a Department of Prosthodontics and Orthodontics, The Affiliated Tai’an City Central Hospital of Qingdao University, Taian, China; b Department of Gastroenterology, University of Health and Rehabilitation (Qingdao Municipal Hospital), Qingdao, China; c Department of Oral and Maxillofacial Surgery, The Affiliated Tai’an City Central Hospital of Qingdao University, Taian, China

*Dear Editor,*

We appreciate the authors’ work in the recent article “Leveraging artificial intelligence for perioperative cancer risk assessment of oral potentially malignant disorders.” This article systematically explores the application prospects and challenges of artificial intelligence (AI) in cancer risk assessment for oral potentially malignant disorders (OPMDs). Research indicates that AI can integrate multidimensional data – including clinical, pathological, and molecular factors – to overcome the subjectivity and limitations of traditional oral epithelial dysplasia (OED) grading, thereby enabling more precise prediction of malignant transformation. Based on this article, we present our own insights.

## Insight 1: The integrative potential of AI in OPMD cancer risk assessment

The article thoroughly examines the potential of AI in assessing cancer risk in OPMDs^[1]^. Currently, OED grading remains the primary method for evaluating malignant transformation risk, but it has significant limitations, including subjectivity, variability between grading systems, and an inability to incorporate all relevant risk factors. AI offers a solution by integrating diverse risk factors – such as demographic data, behavioral risks (e.g., smoking), lesion characteristics, comorbidities, and molecular biomarkers – to provide a more comprehensive and objective risk assessment. For instance, AI models can analyze structured data (e.g., electronic health records) and unstructured data (e.g., clinical images, histopathology slides) to predict malignant transformation probabilities. This integration not only enhances the predictive accuracy of OED grading but also supports personalized treatment decisions. Additionally, AI-driven feature extraction and pattern recognition can automatically identify high-risk features in clinical images, reducing human error. However, most current AI models remain experimental, lacking large-scale clinical validation and full integration into clinical practice. Future research should focus on multicenter validation and clinical utility assessments to ensure model generalizability across diverse populations.

## Insight 2: Clinical implementation pathways and challenges of AI in OPMD surgical management

The article outlines a detailed clinical implementation pathway for AI models in OPMD risk assessment, covering data collection, preprocessing^[2]^, model training, internal/external validation, clinical impact evaluation, and full-scale deployment. Data preprocessing is particularly critical, emphasizing quality assessment and class imbalance correction techniques (e.g., oversampling, data augmentation) to enhance model performance. However, several challenges persist. First, variability in data sources and quality may impair model generalizability – for example, inconsistencies in electronic health record formats across institutions can degrade cross-center validation performance. Second, ethical and legal concerns (e.g., data privacy, algorithmic transparency) must be addressed. Furthermore, clinician and patient acceptance of AI tools is pivotal. The article suggests that education and training can improve clinician understanding, while transparent communication can foster patient trust. Despite these hurdles, the potential benefits of AI – such as refined risk stratification and personalized treatment – justify further exploration and optimization.

## Insight 3: Limitations and future directions in subtype-specific AI models for OPMDs

The article highlights that existing AI models predominantly focus on oral leukoplakia, with limited research on other OPMD subtypes (e.g., proliferative verrucous leukoplakia, erythroplakia). This narrow focus may compromise predictive accuracy for high-risk subtypes. For instance, proliferative verrucous leukoplakia and erythroplakia exhibit significantly higher malignant transformation rates than leukoplakia, yet their distinct clinical and molecular features are often overlooked in current models. Future research should prioritize developing subtype-specific AI models and validating their performance through subgroup analyses. Additionally, integrating molecular biomarkers (e.g., LOH, p53) could further enhance predictive precision, though technical and cost barriers currently limit their incorporation. Proposed improvements include: (1) Multimodal AI systems: Combining clinical, imaging, and molecular data for holistic risk assessment. (2) International collaboration: Expanding datasets to ensure diversity and representativeness across populations. (3) Clinical translation: Bridging the gap between experimental models and real-world application in precision medicine.

Notably, the current AI research landscape disproportionately emphasizes oral leukoplakia, leaving other OPMD subtypes underexplored. Moving forward, the development of multimodal AI systems integrating clinical and molecular markers, alongside global collaborative efforts, will be crucial for advancing AI-driven precision medicine in OPMD management.

## Data Availability

Not applicable.
